# Immune Responses to Tissue-Restricted Nonmajor Histocompatibility Complex Antigens in Allograft Rejection

**DOI:** 10.1155/2017/6312514

**Published:** 2017-01-09

**Authors:** Ankit Bharat, T. Mohanakumar

**Affiliations:** ^1^Department of Surgery, Northwestern Feinberg School of Medicine, Chicago, IL 60611, USA; ^2^Norton Thoracic Institute, St. Joseph's Hospital and Medical Center, Phoenix, AZ 85013, USA

## Abstract

Chronic diseases that result in end-stage organ damage cause inflammation, which can reveal sequestered self-antigens (SAgs) in that organ and trigger autoimmunity. The thymus gland deletes self-reactive T-cells against ubiquitously expressed SAgs, while regulatory mechanisms in the periphery control immune responses to tissue-restricted SAgs. It is now established that T-cells reactive to SAgs present in certain organs (e.g., lungs, pancreas, and intestine) are incompletely eliminated, and the dysregulation of peripheral immuneregulation can generate immune responses to SAgs. Therefore, chronic diseases can activate self-reactive lymphocytes, inducing tissue-restricted autoimmunity. During organ transplantation, donor lymphocytes are tested against recipient serum (i.e., cross-matching) to detect antibodies (Abs) against donor human leukocyte antigens, which has been shown to reduce Ab-mediated hyperacute rejection. However, primary allograft dysfunction and rejection still occur frequently. Because donor lymphocytes do not express tissue-restricted SAgs, preexisting Abs against SAgs are undetectable during conventional cross-matching. Preexisting and de novo immune responses to tissue-restricted SAgs (i.e., autoimmunity) play a major role in rejection. In this review, we discuss the evidence that supports autoimmunity as a contributor to rejection. Testing for preexisting and de novo immune responses to tissue-restricted SAgs and treatment based on immune responses after organ transplantation may improve short- and long-term outcomes after transplantation.

## 1. Introduction

Human leukocyte antigens (HLA) have traditionally been thought to play a dominant role in the development of alloimmunity and allograft rejection [1–4]. Although the effect of HLA matching on improving posttransplant survival is controversial in racially mixed populations, most agree that preexisting donor-specific HLA antibodies (Abs) significantly predispose to hyperacute or acute antibody-mediated allograft rejection (AMR), particularly if the cross-match is positive. This has led to the universal practice of cross-matching prior to solid organ transplantation and, by extension, a reduction in the rate of hyperacute AMR [5]. Nevertheless, acute allograft dysfunction and acute and chronic allograft rejection have remained unaffected [6]. In the context of lung transplantation (LTx), preexisting donor-specific antibodies (DSA) are known to significantly worsen both short- and long-term outcomes [7–13]. Murine and human studies have established the notion that lung-restricted autoimmunity also plays a central role in lung allograft failure at many levels, suggesting that it may represent the common pathogenic pathway of many injury mechanisms which lead to chronic lung rejection [7, 14]. Importantly, the current system of cross-matching cannot detect preexisting immune responses to lung-restricted antigens [15]. In this review, we will discuss recent advancements in defining immune responses to tissue-restricted self-antigens (SAgs)—that is, autoimmunity—and the role of these immune responses in posttransplant allograft survival, focusing specifically on LTx.

## 2. Pathogenesis of Lung-Restricted Autoimmunity (LRA)

It has traditionally been postulated that self-reactive lymphocytes are deleted in the thymus. However, recent data indicate that nonubiquitous antigens present in organs such as the lung, pancreas, and small intestine are not expressed on the thymocytes, and lymphocytes specific to these SAgs do not undergo thymic deletion [16]. CD4^+^CD25^+^Foxp3^+^ regulatory T-cells (Tregs) dynamically suppress these self-reactive lymphocytes against tissue-restricted SAgs [16]. Because the SAgs are normally sequestered, activation of self-reactive lymphocytes is further prevented. LTx recipients and patients with end-stage lung disease undergo many injury-repair cycles that create an inflammatory milieu, which can lead to the expansion of autoreactive lymphocytes. Some mechanisms that have been proposed for this phenomenon include release of the sequestered SAgs, lowering of activation thresholds of self-reactive lymphocytes [17], and epitope spreading [18, 19]. Recruitment of innate immune cells such as monocytes and neutrophils after ischemia-reperfusion can further contribute to this phenomenon. Innate immune cells recognize pathogens using pathogen-recognition receptors (PRRs), which include Toll-like signaling receptors, nucleotide-binding oligomerization domain- (NOD-) like receptors, and retinoic acid-inducible gene- (RIG-) like helicases. However, the PRRs may also cross-react with SAgs released after cellular damage during transplantation (e.g., hyaluronan and heat shock proteins) triggering an immune response [20]. Therefore, the inflammatory cascade that results after transplantation may play an important role in the development of de novo LRA.

Antibodies against donor human leukocyte antigens predispose to LRA. Hachem et al. demonstrated that 70% of all LTx recipients develop LRA within the first three years of transplantation. However, over 96% of recipients with preexisting donor-specific HLA Abs develop LRA [21]. It has been postulated that the donor-specific HLA Abs can cause lung injury and inflammation [22], which can expose otherwise-sequestered SAgs [23–25]. Similarly, acid aspiration from gastroesophageal reflux disease can lead to lung injury [26] and is a known risk factor for chronic rejection [27–29]. Hence, lung allografts are susceptible to several injury mechanisms that can cause local inflammation [14, 30, 31] and increase the risk of lung-restricted autoimmunity [12, 32]. Tregs are known to suppress both inflammation and immune responses by effector lymphocytes [33–35] by inhibiting cytokine production and proliferation of effector cells [36–39]. Loss of Tregs is associated with both acute and chronic lung allograft rejection [36–41]. Therefore, it stands to reason that an injury mechanism that reveals the sequestered SAgs to the host immune system, combined with loss of Tregs, may lead to further activation of self-reactive lymphocytes and development of tissue-restricted autoimmunity.

We have previously shown that respiratory viruses can induce loss of Tregs in murine models. Specifically, draining lymph nodes of murine recipients of orthotopic tracheal transplantation prompted apoptosis in Tregs after infection of the airway with Sendai virus [42, 43]. This is a transient effect, and Tregs levels return to baseline upon clearance of the viruses. Infection of tracheal epithelial cells with Sendai virus in vitro was found to mediate Treg apoptosis through Fas-FasL interactions. It is of interest that viruses are often associated with a variety of autoimmune diseases [24, 44]. In this context, we demonstrated that LTx recipients who develop respiratory viral infections demonstrate a transient loss of Tregs [42, 45]. Interestingly, if these recipients had a preexisting lung injury mechanism (e.g., donor-specific HLA Abs or gastroesophageal reflux), they are at increased risk of developing de novo lung-restricted autoimmunity.

Both murine and human LTx recipients infected with respiratory viruses show increases in FasL in the bronchoalveolar lavage fluid. We therefore tested whether lung injury and concomitant loss of Tregs in wild-type hosts would lead to LRA. Immunocompetent mice were injected with either hydrochloric acid or Abs to MHC class I, and Tregs were depleted by either murine parainfluenza Sendai virus (in wild-type mice) or diphtheria toxin (in Foxp3-DTR mice) [45]. This resulted in the development of both cellular and humoral immunity against lung-restricted SAgs. Lung injury with the MHC Abs, hydrochloric acid without depletion of Tregs, or depletion of Tregs without lung injury did not trigger autoimmunity. In human subjects, patients with cystic fibrosis and idiopathic pulmonary fibrosis are predisposed to ongoing lung injury and respiratory infections, and individuals with these diseases have the highest prevalence of LRA before LTx compared to patients with other diseases (e.g., emphysema and alpha-1-antitrypsin deficiency) [46–50]. The evidence from murine and human models suggests a “two-hit” mechanism for the development of lung-restricted autoimmunity wherein both lung injury and loss of Tregs are essential.

Recent data show that LTx recipients develop exosomes containing lung-restricted antigens, and this might be a biomarker for allograft rejection [51]. However, detection of exosomes, both in serum and in bronchoalveolar lavage fluid, raises the possibility that these exosomes might be involved in the development of lung-restricted autoimmunity. It is possible that the underlying lung injury mechanism leads to the formation of exosomes that incorporate lung-restricted SAgs as well as several immunoregulatory microRNAs, which are released into the circulation and facilitate the generation of autoimmunity due to their immunogenic potential [52–55]. The mechanisms that lead to exosome formation and how these mechanisms may trigger autoimmunity remain to be elucidated.

## 3. Role of Tissue-Restricted Abs in Organ Transplantation

### 3.1. Lung Transplantation

Compared with other types of solid organ transplantation, LTx has the lowest survival rate. Development of primary graft dysfunction (PGD) within the first 72 hours and chronic allograft rejection within six months are the two predominant causes of this poor outcome [6, 56, 57]. Intriguingly, PGD has emerged as one of the strongest risk factors for chronic lung allograft rejection. We have previously demonstrated that PGD is associated with a robust inflammatory response that promotes development of alloimmunity, autoimmunity, and chronic rejection [3, 11]. PGD has been thought to be the result of ischemia-reperfusion injury [58, 59], but this hypothesis conflicts with the recent observation that ischemic time may not correlate with PGD development [60, 61]. In other words, it is not uncommon to observe high-grade PGD development in lung allografts with very short ischemic times, or grafts with more than 8 hours of ischemia that do not develop PGD. It is also noteworthy that several histological hallmarks of PGD, such as alveolar edema, capillaritis, hyaline membrane formation, and neutrophil infiltration [62, 63], are similar to features observed in antibody-mediated rejection (AMR) after LTx, which raises the possibility of PGD being caused by some form of preexisting Abs [64–68].

As previously discussed, LTx recipients, like other solid organ transplant recipients, are rigorously screened for donor-specific HLA Abs. Nevertheless, we detected complement deposition and increases in soluble complement in the allograft biopsies and bronchoalveolar lavage fluid, respectively, in patients with PGD who did not have Abs against HLA [11]. Similar findings have been noted by Westall et al., who found complement deposition in human transbronchial allograft biopsies obtained from patients who developed PGD after LTx [69]. Further longitudinal analysis revealed that about 30% of patients undergoing LTx have preexisting Abs against the lung-restricted antigens collagen type V (Col V), collagen type I, and k-alpha 1 tubulin (K*α*1T). These lung-restricted SAgs strongly predispose patients to PGD, development of de novo alloimmunity, and chronic lung rejection [11, 12, 70]. In fact, the presence of all three Abs before transplantation was associated with PGD by over 7-fold magnitude [8–11].

Because a number of clinical factors can confound the association between preexisting Abs to lung SAgs and PGD, we tested whether these two variables are mechanistically linked using the murine model of unilateral LTx [71]. In this recent study, recipients were passively given one or more Abs to lung-restricted antigens before transplantation of syngeneic lung grafts. Each of the Abs demonstrated a dose-dependent graft dysfunction of the syngeneic grafts. Interestingly, preexisting LRA led to epitope spreading wherein administration of Col V Abs induced de novo K*α*1T Abs after LTx and vice versa. We further used allogeneic LTx to investigate whether preexisting LRA could prevent development of tolerance. Using MR1 and CTLA4-Ig, tolerance can reliably be achieved toward MHC-mismatched lung allografts. However, preexisting LRA prevented tolerance development and led to dose-dependent development of donor-specific alloimmunity and chronic lung allograft dysfunction. The same held true for de novo LRA [72]. Similar to preexisting LRA, de novo development of LRA after LTx can lead to rejection of a syngeneic lung allograft and can prevent allotolerance toward MHC-mismatched lungs. Development of PGD associated with preexisting LRA has also been demonstrated in the rat LTx model. In that study, the authors administered Col V Abs in rats prior to syngeneic graft transplantation. Rats who received the Col V Abs developed a syndrome of PGD [12]. The authors demonstrated that lung allografts with PGD associated with Col V Abs demonstrated both Ab and complement deposition.

Other reports have confirmed the presence of Col V-specific T-cells after allogeneic rat LTx [9]. When adoptively transferred into recipients of syngeneic lung grafts, these Col V-specific T-cells induced rejection [73]. We previously found that expansion of IFN-*γ*-producing, Col V-specific Th-1 cells together with reduction in IL-10 secreting T-cells is associated with development of chronic lung allograft rejection [8, 74, 75]. In an experimental model of chronic lung allograft rejection, adoptive transfer of lymphocytes with high levels of IL-17 and IL-23 gene transcripts from Col V-sensitized mice induced histological lesions of obliterative airway disease observed in chronic lung allograft rejection after syngeneic LTx [9].

Another non-HLA antigen associated with lung allograft rejection is MHC class I-related chain A (MICA). MICA is a glycoprotein expressed on cellular membrane which, when expressed, indicates cellular stress and triggers a variety of immune effector mechanisms [76, 77]. MICA can bind to the immune-receptor NKG2D and provides costimulatory signal for the activation of natural killer (NK), CD8^+^ T, and *γδ* T-cells after LTx [78]. Abs to MICA after solid organ transplantation have been associated with chronic rejection [79]. It appears that anti-HLA often precedes the development of anti-MICA, and peak titers of anti-MICA are present at the time of clinical diagnosis of chronic lung allograft rejection [80].

The importance of de novo LRA after transplantation has been established in clinical settings. In an important study by Hachem et al., more than 96% of LTx recipients with preexisting donor-specific HLA Abs developed de novo LRA within three years of LTx and were strongly predisposed to development of chronic rejection [21]. However, Ab-directed therapy was only effective in reversing the increased risk of chronic rejection if it cleared LRA. Patients who cleared HLA Abs but had persistent LRA demonstrated the same risk of developing chronic rejection as those with both HLA Abs and LRA, suggesting that LRA is an important contributor to chronic rejection.

### 3.2. Heart Transplantation

Over 40% of patients with cardiac allografts develop chronic rejection—which is manifested by vasculopathy—within 5 years [81]. Th17 cells have been shown to mediate a proinflammatory response leading to chronic allograft vasculopathy (CAV) in the absence of Th1 response [82]. T-cell autoreactivity against cardiac myosin, a SAg present in the heart, can develop in the absence of alloimmune responses and is associated with the development of chronic CAV [83, 84]. Humoral immunity has also been implicated in the pathogenesis of CAV [85, 86]. Abs against vimentin, a cytoskeleton protein, independently increase the risk of CAV and accelerate its course [83, 87, 88]. Additionally, humoral immunity to mismatched MICA has been reported to contribute to the immunopathogenesis of both AMR and CAV after heart transplantation [89, 90].

### 3.3. Kidney Transplantation

Chronic allograft nephropathy is the predominant cause of kidney graft failure [91]. Although alloimmune responses are important in renal allograft rejection, clinically refractory rejection may be associated with Abs against non-HLA antigens. Abs against angiotensin II type 1 (AT1) receptor [92] have been shown to increase the risk for refractory allograft rejection. Other non-HLA antigens that have been shown to play a role in kidney allograft rejection include perlecan, Col IV, Col VI, and the glomerular basement membrane protein, agrin [93, 94]. Recent studies have also suggested a role for antivimentin in the development of chronic renal rejection [95]. Transplant glomerulopathy (TG) is another form of renal allograft dysfunction that can affect over 20% of patients within 5 years of transplantation [93]. TG usually results from humoral injury to the endothelial cells [93], and both HLA and non-HLA Abs have been shown to play roles in its development. Non-HLA Abs include those against AT1, Col IV, fibronectin, MICA, and agrin. Antiglomerular basement membrane Abs against heparan sulphate proteoglycan agrin can also predispose to TG [96].

### 3.4. Liver Transplantation

Chronic rejection after liver transplantation manifests itself as allograft fibrosis. Recurrence of hepatitis C virus (HCV) is universal after orthotopic liver transplantation (OLT) in HCV-infected recipients [97]; this recurrence is associated with the remodeling of extracellular matrix and its components, including collagen (which promotes fibrogenesis). This process can generate Abs against liver collagen and further augment this process [98–101]. Increased levels of Abs against Col II, Col IV, and vimentin are found in patients with liver fibrosis before transplantation and in patients who develop allograft fibrosis. Patients with native liver and allograft fibrosis also demonstrate significantly higher T helper 2 (Th2) and T helper 17 (Th17) cytokine levels and lower T helper 1 cytokine levels than recipients without fibrosis. Our previous results have also demonstrated that, in HCV-infected patients, levels of Abs to extracellular matrix protein positively correlate with liver fibrosis, which is associated with a predominant Th2 and Th17 cytokine profile [98]. Taken together, these results suggest that development of liver-restricted autoimmunity might play a role in liver allograft fibrosis following OLT.

### 3.5. Mechanism of Action of Lung-Restricted Abs

The precise mechanisms of action of Abs to lung-restricted SAgs remain unknown—a hurdle which has resulted in inconsistent diagnosis of humoral allograft rejection mediated by such Abs. For example, complement deposition is used as a marker of humoral rejection even though it has not been conclusively shown that LRA can activate complement. We recently reported a case series of human LTx recipients in which a form of hyperacute and acute humoral rejection was caused by preexisting LRA [62]. This was associated with deposition of LRA and complement on the allograft. Furthermore, both types of rejection were successfully treated using Ab-directed therapy, including intravenous immunoglobulin and plasmapheresis. This suggests that the acute effects of LRA may indeed involve complement activation. Nevertheless, patients with LRA-mediated lung injury demonstrate neutrophil recruitment, but whether the newly arriving neutrophils play a mechanistic role remains unclear. Additionally, several immune cells including monocytes, macrophages, dendritic cells, neutrophils, and NK cells have Fc receptors. LRA ligation with the cognate antigens can potentially activate these immune cells and mediate their pathogenic effects. These mechanisms must be investigated in future studies.

In lung allografts, respiratory epithelium may be the primary target of a recipient's immune system. Abs to K*α*1T, an epithelial cell surface gap junction protein, cause upregulation of profibrotic growth factors [10]. Lipid rafts may also play a critical role in the surface ligation of K*α*1T Abs to their antigens on the surfaces of airway epithelial cells [102, 103]. Normal human bronchial epithelial cells demonstrated upregulation of profibrotic growth factors (e.g., VEGF, HGF, and TGF-*β*), after ligation with K*α*1T Abs. Additionally, hypoxia-inducible factor (HIF-1*α*) was increased as a consequence of K*α*1T Ab ligation on airway epithelial cells. Suppression of HIF-1*α* reversed the production of profibrotic growth factors upon ligation with K*α*1T Abs. These studies suggest that LRA promotes fibrosis and can contribute to chronic rejection.

## 4. Current Limitations and Future Directions

A wealth of recent literature convincingly supports the role of tissue-restricted Abs in allograft dysfunction and rejection, particularly in the context of LTx. Both human and murine studies have mechanistically linked LRA with lung allograft rejection; however, several questions remain unanswered. First, the treatment thresholds for LRA in LTx recipients are unknown. If a recipient is positive for donor-specific HLA Abs before transplantation, the donor can be excluded or the recipient can be desensitized. However, because these SAgs are nonpolymorphic and are present in all humans, the presence of pretransplant LRA poses a clinical dilemma pertaining to management. LRA likely become pathogenic above a certain titer, but perhaps their pathogenicity is determined by the expression of cognate SAgs in the donor lungs. Because donor lungs are exposed to multiple injury mechanisms (e.g., brain death, mechanical ventilation, variable levels of warm and cold ischemia, and pneumonia), the levels of expression of SAgs may vary. Hence, equivalent titers of LRA could cause varying effects in different donor lungs. Second, although the testing of these Abs is based on ELISA, there are no commercial tests presently available for clinical application and it is unlikely that they will be made available until Ab testing is widely adopted. Third, LRA were detected based on lung epithelial and endothelial cells lines, and it may be possible that patients with end-stage lung disease might have Abs against additional undetected SAgs. Therefore, testing of the LRA present at a given moment may be insufficient to prevent PGD and chronic lung allograft rejection. Fourth, the mechanisms of action of LRA remain unclear: although complement deposition is linked to LRA-associated lung dysfunction, it is unknown whether complement activation is indeed necessary for the pathogenic effects of LRA. Because lung myeloid cells such as macrophages, monocytes, neutrophils, and dendritic cells express Fc receptors, LRA might directly activate the immune cells and mediate lung rejection. It is important to elucidate these mechanisms, as the mechanism of action will inform the treatment selected. Lastly, it remains unclear whether expansion of self-reactive T-cells plays a role in lung allograft rejection. Further investigation is imminently required to answer these questions, as targeting lung-restricted autoimmunity represents a clinically applicable therapeutic avenue with the potential to significantly improve outcomes in LTx and transplantation of other solid organs.

## Glossary

Abs: Antibodies
AMR: Antibody-mediated rejection
AT1: Angiotensin II type 1
CAV: Chronic allograft vasculopathy
Col V: Collagen type V
HLA: Human leukocyte antigens
K*α*1T: k-alpha 1 tubulin
LRA: Lung-restricted autoimmunity
LTx: Lung transplantation
MHC: Major histocompatibility complex
MICA: MHC class I-related chain A
NOD: Nucleotide-binding oligomerization domain
OLT: Orthotopic liver transplantation
PGD: Primary graft dysfunction
PRR: Pathogen-recognition receptors
RIG: Retinoic acid-inducible gene
SAgs: Self-antigens
TG: Transplant glomerulopathy
Tregs: CD4^+^CD25^+^Foxp3^+^ regulatory T-cells.
